# Spinopelvic parameters, saggital balance and compensatory mechanics in younger and older adults with scoliosis

**DOI:** 10.1186/1748-7161-9-S1-O40

**Published:** 2014-12-04

**Authors:** Larry Ian Cohen

**Affiliations:** 1 Postural Physiotherapy, Sydney, Australia

## Background

Saggital balance is an important factor in postural alignment that has been linked to back pain, dysfunction and quality of life. It is reportedly even more important than coronal curve values. The Scoliosis Research Society (SRS) has recently proposed saggital balance threshold values for evaluating saggital vertical axis (SVA) and lumbopelvic compensations as defined by pelvic incidence–lumbar lordosis (PI-LL) (±50mm and± 10°, respectively).

## Aim

To evaluate SVA and PI-LL with the current gold standard (low dose EOS x-ray scans)(2) in younger and older patients with scoliosis.

## Design

Retrospective cohort study of 27 consecutive adult patients (45 ±19 years) with scoliosis (coronal cobb>10°) who underwent EOS scanning were separated into a younger (<45 years, n=14) and older (≥45 years, n=13) group.

## Methods

Fisher’s exact tests were used to evaluate differences in the prevalence of SVA and PI-LL threshold deformities in patients younger and older than 45 years old.

## Results

38% of the older group and 0% of the younger group exceeded the SVA threshold of +50mm (p=0.02). No difference between the younger and older group for the prevalence of PI-LL mismatch compensation was found (36% vs. 62%’ p = 0.25). However, 100% of the younger mismatch subgroup was below -10 degrees and 67% of the older mismatch subgroup exceeded 10 degrees (p = 0.02).

## Conclusions

This study demonstrates that patients use compensations to maintain upright sagittal balance. Older patients more frequently exhibit decompensated positive sagittal balance beyond the 50mm SVA threshold. Whilst there were no differences between the age groups with respect to the proportion of patients who cross the PI-LL threshold, younger patients may compensate by anterior pelvic tilt and lumbar hyperlordosis whereas older patients may compensate by posterior pelvic tilt and lumbar hypolordosis.

Prospective studies are needed to examine the aetiology, pathogenesis and pathomechanisms of compensations and if further compensatory changes occur as patients age(3). Physiotherapists who see patients many years before they attend surgical assessments may be able to play an important role in determining, predicting compensations, utilising the information for treatment, monitoring and even preventing the ramifications of sagittal balance failure

